# Assessing the combined toxic effects of metaldehyde mollucide

**DOI:** 10.1038/s41598-023-32183-6

**Published:** 2023-03-25

**Authors:** Oksal Macar, Tuğçe Kalefetoğlu Macar, Kültiğin Çavuşoğlu, Emine Yalçın, Ali Acar

**Affiliations:** 1 Department of Food Technology, Şebinkarahisar School of Applied Sciences, Giresun University, Giresun, Turkey; 2 Faculty of Science and Art, Department of Biology, Giresun University, Giresun, Turkey; 3 Department of Medical Services and Techniques, Vocational School of Health Services, Giresun University, Giresun, Turkey

**Keywords:** Cell biology, Genetics, Molecular biology

## Abstract

The excessive use of metaldehyde in agriculture to combat mollusks endangers both the environment and non-target organisms. The aim of this study is to investigate the toxicity caused by metaldehyde in *Allium* *cepa* with the help of physiological, cytogenetic, biochemical and anatomical parameters. Also, DNA fragmentation caused by metaldehyde in root tip cells was measured by the "Comet Assay" method. The control group was germinated with tap water and the application groups were germinated with 20 mg/L metaldehyde, 40 mg/L metaldehyde, 100 mg/L metaldehyde and 200 mg/L metaldehyde for 72 h. The results of the physiological parameters showed that metaldehyde had a growth-limiting effect in *A.* *cepa*, depending on the application dose. According to root elongation levels, the EC_50_ (effective concentration) value for metaldehyde was 60.6 mg/L in *A.* *cepa*. As the treatment dose increased, the incidence of micronucleus and chromosomal aberrations gradually increased while mitotic index decreased. Metaldehyde exposure induced damages such as sticky chromosome, fragment, unequal distribution of chromatin, reverse polarization, bridge, and multipolar anaphase. In addition, metaldehyde caused cell damage in epidermis and cortex, thickening of the cortex cell wall and flattened cell nucleus in root meristem. Increasing doses of metaldehyde application also increased malondialdehyde levels, superoxide dismutase and catalase activities. As a result, it has been determined that the toxicity of metaldehyde in plants is versatile and the *A.* *cepa* test material is a suitable biological indicator to determine this toxicity.

## Introduction

Pesticides are chemical compounds that enable the reduction of agricultural losses, increase in yield as well as abundant and inexpensive food production. The development of pesticides, including herbicides, fungicides, insecticides and mollucides, has increased gradually since World War II. Since then, the population boom in the twentieth century has forced an increase in food production, and numerous advances in agricultural technology have been accompanied by the rise in pesticide use^1^. However, pest resistance that develops due to the increasing administration of pesticides causes both economic losses and necessitates the use of more and more toxins in fields^2^. While only 1% of the 3 million tons of pesticides used in the world each year are effective to protect target crops from pests, the rest accumulate in the environment and cause health problems for non-target species^3,4^.

Mollusks such as slugs and snails are perilous pests not only to crops, but also to a wide variety of agricultural products, including vegetables, ornamental plants, paddy and oilseeds, especially in the rainy seasons^5^. Metaldehyde (C_8_H_16_O_4_ = 2, 4, 6, 8-tetramethyl-1, 3, 5, 7-tetraoxacyclooctane) is a mollucide that has been practiced to exterminate gastropods since the 1940’s^6^. As a polar dry alcohol, it is produced by the polymerization of acetaldehyde. Once taken into the body, it causes the mollusks to secrete excessive mucus and dry out completely^7^. This chemical compound and residues of which could be found in harvested fruits and vegetables, is capable of entering the bloodstream through digestion in humans and other non-target creatures, causing poisoning^8^. According to studies focused on mammals, pets and wild animals, metaldehyde has been classified as a moderately toxic compound^9^. It also has neurotoxic effects and causes vomiting, tachycardia, tachypnea, ataxia, tremors and seizures that can result in death^10^. Booze and Oehme^11^ mentioned that metaldehyde toxicity observed in dogs is due to the direct action of metaldehyde rather than acetaldehyde produced by gastric hydrolysis of metaldehyde. In the literature, the Lethal Dose 50 (LD_50_) of metaldehyde in dogs is greater than 600 mg/kg body weight ^11^, while the Lethal Concentration 50 (LC_50_) of metaldehyde in climbing bass (*Anabas* *testudineus*) is 239 mg/L^12^. Concerns about metaldehyde pollution are increasing due to its very long half-life (water; 17 and soil; 223 days) and low biodegradability^13^. In addition, it is extremely difficult and costly to remove metaldeyde from water^14^.

*Allium* *cepa* has become one of the most used model organisms in plant-based toxicity studies due to its large sized and small number of chromosomes that can be easily seen under light microscopy, easy accessibility, low cost, reliability and high correlation with other test systems^15,16^. *Allium* assay has been used for many years to elucidate the genotoxic effects of pesticides in living organisms^17,18^.

Although the toxic effects of metaldehyde have been studied in different organisms before, there is no comprehensive study investigating its physiological, cytogenetic, biochemical and genotoxic effects on plants. The aim of this study is to investigate the toxic effects of metaldehyde mollucide in all aspects in *A.* *cepa* test material.

## Materials and methods

### Preparation of materials and experimental setup

In this study, metaldehyde (CAS Number: 9002-91-9/1 KG), a product of the Sigma-Aldrich company, was used. *A.* *cepa* bulbs (n = 16) purchased from a local grocery store were selected to be approximately equal in weight (7.10–9.00 g). All procedures were conducted in accordance with the guidelines. The bulbs were thoroughly washed under running tap water to remove dust. The brown scales on the outermost part of the bulbs were peeled off and the old roots were cut away. *Allium* bulbs were then divided into five groups, consisting of a control and four treatment groups. The control group was kept in glass tubes filled with tap water so that the basal plates of the bulbs touched water throughout the experimental process. Treatment groups 1, 2, 3 and 4 were exposed to aqueous solutions of 20 mg/L metaldehyde, 40 mg/L metaldehyde, 100 mg/L metaldehyde and 200 mg/L metaldehyde solutions, respectively. All applications were carried out in a dark chamber at room temperature for 72 h.

### Analysis of growth parameters

Once the experiment was terminated, root elongation was assessed by measuring the lengths of the adventitious roots that grew during the experiment by a ruler. The EC_50_ value, the point indicating 50% of the growth, was determined using the root length measurements of five different groups. The bulbs were weighed at the end of the experiments. To determine the weight gain, the difference between the final weight and the weight recorded before the experiments was taken for each bulb. The emergence of adventitious roots from the basal plate of the bulbs was considered “germination” to calculate the germination percentage (Eq. 1)^19^. The relative injury rate (RIR) was calculated using the formula (Eq. 2).

1 $${\text{GP }}\left( \% \right) \, = \, \left( {{\text{Number \, of \, the \, germinated \, bulbs }}/{\text{ Total \, number \, of \, the \, bulbs}}} \right) \, \times \, { 1}00$$

2 $${\text{RIR}} = \, \left( {\% {\text{GP \, in \, control \, group }} - \, \% {\text{GP \, in \, each \, group}}} \right) / \left( {\% {\text{GP\, in \, control}}} \right)$$

### Analysis of genotoxicity parameters

The roots were decapitated to perform the analysis of cytogenetic parameters. The frequencies of both CAs and MN incidences were determined according to the method of Staykova et al.^20^. Root tips were fixed using Clarke’s fixator (glacial acetic acid/ethanol = 3:1) and washed thoroughly with distilled water. Root tips were hydrolyzed at 60 °C using 1 N hydrochloric acid for 12 min. Hydrolyzed root tips were washed again with distilled water before being stained with 1% acetocarmine for 24 h at room temperature. To prepare examination slides, root tips were squashed between the slide and coverslip with a drop of 45% acetic acid solution. Ten slides from each treatment were observed under a research microscope at 400× magnification. The method of Fenech et al.^21^ used to evaluate MN frequency. CAs and MN frequencies were calculated by examining 100 cells from each slide (1000 cells for each treatment). On the other hand, MI was determined by examining 100 cells from each slide (10,000 cells for each treatment). MI was calculated as the ratio of cells in the mitotic phase to the total number of cells observed.

### Comet assay (single-cell gel electrophoresis)

For alkaline single-cell gel electrophoresis, the protocol of Chakraborty et al.^22^ was performed. The roots were quickly crushed with a raster tool in 400 μL of tris buffer (cold, 0,4 M, pH 7.5) and a mixture of 1:1, 1% low melting point agarose (LMPA). Nuclear suspension and 1% LMPA in phosphate-buffered saline (PBS) were added to the 1% NMPA pre-coated slides. The coverslip was gradually removed after the LMPA gelling stage. For 15 min to a horizontal gel electrophoresis tank with a cooled and fresh electrophoresis buffer, with 4 min of electrophoresis at 4 °C of 0.7 V/cm (20 V, 300 mA), the embedded nuclei slides in the LMPA were transferred. Slides were rinsed three times with filtered water and neutralized with tris buffer (0.4 M Tris, pH 7.5). The nuclei were stained for 5 min with ethidium bromide after immersion in cold water for 5 min. To remove any remaining stain, the preparations were washed with cold water and the coverslip was sealed. These steps were taken with low light in order to avoid DNA degradation and were examined with a fluorescence microscope. Comets were analyzed with Comet Assay software version 1.2.3b^23^ with the parameters of tail DNA length. A total of 1.200 cells were analyzed for each group, 200 in each bulb for DNA damage. The extent of DNA damage was scored from 0 to 4 depending upon the level of DNA damage. The cells were classified into five categories based on tail DNA length, ranging from zero to four, according to Collins^24^. The total DNA damage per group, expressed as arbitrary units, was calculated using Eq. (3).

3 $${\text{Arbitrary\, unit }} = \sum\nolimits_{{{\text{i}} = 0}}^{4} {{\text{Ni}} \times {\text{i}}} \,$$

(i is the degree of damage (0, 1, 2, 3, 4), Ni is the number of cells in i degree).

### Analysis of SOD and CAT activities

Analysis of SOD and CAT activities was performed using the standard extraction method^25^. A 0.2 g root sample was homogenized in 5 mL of cold 50 mM sodium phosphate buffer (pH 7.8) using a cold mortar and pestle. After the homogenate was centrifuged at 10,000 rpm for 20 min, the supernatant was used to determine the SOD and CAT activities.

The method proposed by Beauchamp and Fridovich^26^ was used to evaluate the activity of SOD enzyme. SOD enzyme activity was determined by measuring the reduction of nitro blue tetrazolium (NBT) spectrophotometrically at 560 nm. Results of SOD enzyme activity were expressed as units per milligram fresh weight (Unit/mg fresh weight).

The method mentioned by Beers and Sizer^27^ was used to evaluate the activity of CAT enzyme. CAT enzyme activity was determined by measuring the enzymatic breakdown of H_2_O_2_ spectrophotometrically at 240 nm. Results of CAT enzyme activity were expressed as OD240 nm min/g fresh weight.

### Analysis of MDA levels

At the end of the 72nd h, MDA levels of *A.* *cepa* root samples of groups were analyzed using the method proposed by Unyayar et al.^28^. A 0.5 g root sample was homogenized in a 5% trichloroacetic acid (TCA) solution with a mortar and pestle. The obtained homogenates were centrifuged at 12,000 rpm for 14 min at room temperature. Supernatant and 20% TCA–0.5% thiobarbituric acid (TBA) solution were mixed in the same amounts in a test tube. Test tubes with mixtures were heated in a hot water bath at 98 °C for 23 min in a hot water bath. At the beginning of 24th min, the test tube was put in an ice bath to stop the reaction. Cooled mixtures were centrifuged at 10,000 rpm for 5 min at room temperature. Supernatant was taken and its absorbance at 532 nm and 600 nm was measured using a spectrophotometer (Shimadzu 1240 UV–VIS spectrophotometer).

### Anatomical observations

Root tips were cut about 1 cm long, washed in distilled water, placed between foam material and cross-sectioned with a sterile razor blade. Sections were placed on slides and stained with 5% methylene blue for 2 min. Detection of root meristem cell damage was made under the IRMECO IM-450 TI model research microscope at 200× magnification and photographed^29^.

### Statistical analysis

Statistical analysis was performed using the SPSS Statistics 22 (IBM SPSS, Turkey) package program. Data are shown as mean ± standard deviation (SD). Statistical significance between the data was determined using one-way analysis of variance, “One-way ANOVA” and “Duncan” tests. When p < 0.05, it was considered statistically significant.

## Results and discussion

Physiological analyses enabled us to evaluate the macroscopic effects of different metaldehyde doses in *A.* *cepa* (Table 1). While the germination percentage of the control group was 100%, the germination percentage decreased as the metaldehyde dose increased in the metaldehyde applied groups. Therefore, the most prominent drop in the germination percentage of the treatment groups was observed in MA-200 mg/L. Treatment 1, exposed to a lower metaldehyde concentration, had a lower relative injury rate (0.06). Relative injury rates of MA-20 mg/L, MA-100 mg/L and MA-200 mg/L were 0.16, 0.30 and 0.42, respectively. Metaldehyde also inhibited the root growth of the groups depending on the application dose. Root elongation was reduced by 23% in MA-20 mg/L and 72% in MA-200 mg/L compared to the control. The EC_50_ value is a useful parameter for selecting test concentrations to perform genotoxicity tests^30^. In this study, EC_50_ value for metaldehyde on *A.* *cepa* was determined as 60.6 mg/L. This result confirms that the concentrations selected in the study are suitable for genotoxicity and toxicity tests. Metaldehyde-related deceleration of weight gain was statistically significant in all groups, similar to inhibition of root elongation. Compared to the control group, the bulb weight of the groups exposed to metaldehyde was reduced by 1.3, 1.8, 2.4 and 4.7 times, respectively. Although there are many studies in the literature on the toxicity of metaldehyde in non-target organisms such as ducklings, dogs, cats and macro-invertebrates^31–34^, to the best of our knowledge, this is the first study to reveal metaldehyde toxicity in *A.* *cepa*. Although Rolph et al.^14^ refered metaldehyde doses up to 1 µg/L are environmentallyrelevantconcentrations, higher doses were used in this study in order to observe the acute toxic effects of metaldehyde in a short time period under laboratory conditions. On the other hand, Ester and Nijenstein^35^ mentioned that metaldehyde application to perennial ryegrass (*Lolium* *perenne*) at rates exceeding 320 g per kg seed had a phytoxic effect by reducing germination. Roots are the main gateways for the entrance of metaldehyde into a plant during germination^36^. Therefore, it is not surprising that the first place where chemical damage to the plant can be morphologically observed is the roots. It is thought that the decrease observed in physiological parameters as a result of metaldehyde exposure may be due to the fact that metaldehyde reduces the uptake of water and mineral substances from the roots and the division of root cells. Indeed, there is some information in the literature that pesticides promote a decrease in physiological parameters by reducing the water and mineral substance intake of plant roots or by inhibiting root cell division^37,38^.

**Table 1:** Effects of metaldehyde treatments on physiological parameters.

Groups*	Germination percentage (%)	Relative injury rate	Root length (cm)	Weight gain (g)
Control	100	0.00	8.56 ± 1.17^a^	4.42 ± 0.96^a^
MA-20 mg/L	94	0.06	6.55 ± 1.91^b^	3.38 ± 0.85^b^
MA-40 mg/L	84	0.16	4.78 ± 1.21^c^	2.50 ± 0.76^c^
MA-100 mg/L	70	0.30	3.82 ± 2.42^d^	1.85 ± 0.64^d^
MA-200 mg/L	58	0.42	2.38 ± 0.55^e^	0.94 ± 0.58^e^

The means shown with different superscript letters (a–e) in the same column were significant at p < 0.05.

*MA* metaldehyde.

*Data were shown as mean ± SD. 50 bulbs were used for germination percentage and 10 bulbs were used for root length and weight gain.

**Figure 1:**
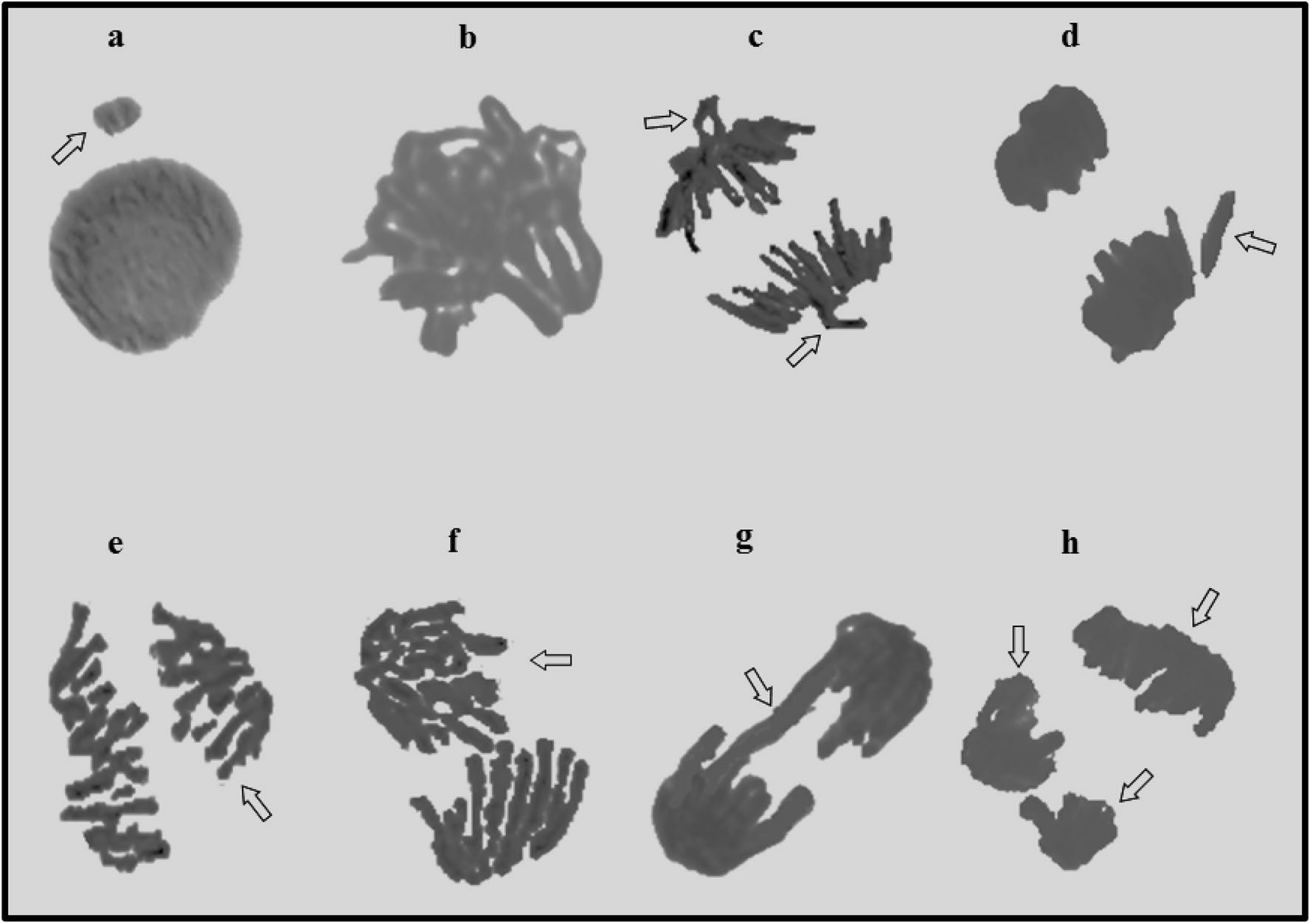
Metaldehyde induced chromosome aberrations. Micronucleus (**a**), sticky chromosome (**b**), vagrant chromosome (**c**), fragment (**d**), unequal distribution of chromatin (**e**), reverse polarization (**f**), bridge (**g**), multipolar anaphase (**h**).

In order to determine the genotoxic effects of metaldehyde on *A.* *cepa* root meristem cells, the MI values and frequencies of MN and CAs were investigated (Table 2) (Fig. 1). As an indicator of cell proliferation rate, MI provides valuable information about the toxic and genotoxic effects of chemicals. Increasing metaldehyde doses decreased the MI values of the MA-20 mg/L group (24%), the MA-40 mg/L group (37%), the MA-100 mg/L (48%) and the MA-200 mg/L group (57%). Application of metaldehyde reduced the successful mitosis rate during the germination process. The results of MI were well-correlated with our growth parameters, particularly with decreases in root elongation and weight gain. Similarly, Asita and Hatane^39^ reported that the application of a mixture of metaldehyde, 30 g/kg and carbaryl, 20 g/kg on *A.* *cepa* reduced the MI value. The determination of MN has a very important role in investigating the toxicity and genotoxicity of pesticides^40^. Contrary to MI values, MN frequencies on *A.* *cepa* meristem cells increased gradually as a result of metaldehyde treatment in dose dependent manner (Fig. 1a). Among the metaldehyde treatments, the highest MN frequency was observed in the MA-200 mg/L treatment (45.50 ± 2.90) and the lowest MN frequency was observed in the MA-20 mg/L treatment (17.90 ± 2.69). MN formation may be the result of breaks in microtubules and chromosomes or single-strand breaks in DNA^41,42^. Although no previous report was found about metaldehyde-induced MN formation, many researchers considered increasing MN frequencies as a sign of the genotoxic effect of pesticides on plants^17,43–45^. In parallel to the MN frequencies, the frequencies of all CAs types were also gradually increased by increasing doses of metaldehyde applications. Frequencies of CAs caused by metaldehyde were sorted from high to low as follows; sticky chromosome (Fig. 1b), vagrant chromosome (Fig. 1c), fragments (Fig. 1d), unequal distribution of chromatin (Fig. 1e), reverse polarization (Fig. 1f), bridge (Fig. 1g) and multipolar anaphase (Fig. 1h). In accordance with our study, Asita and Hatane^39^ reported that sticky chromosomes were most seen CAs as a result of metaldehyde/carbaryl mixture application. Sticky chromosomes were the most possible CA formation in the case of deterioration in DNA^46^. Sticky chromosomes can result from the adhesion of chromosomal proteins or defects in nucleic acid metabolism in the cell, or the dissolution of the protein that covers the DNA^18^. On the other hand, vagrant and laggard chromosomes are indicators of spindle malfunction, while fragments and bridges are attributed to clastogenicity^47^. Considering that bridge and fragment aberrations are directly related to MN formation^48^, the increases in these aberrations paralleled the results of MN in this study. Drastic increases in the frequencies of MN and CAs induced by metaldehyde clearly demonstrated the genotoxic properties of metaldehyde. These increased MN and CAs also caused retardation in MI and growth parameters by reducing the rate of successful cell division in the mitosis phase.

**Table 2:** Genotoxicity induced by metaldehyde in root tip meristem cells of *A.* *cepa*.

Damages	Control	MA-20 mg/L	MA-40 mg/L	MA-100 mg/L	MA-200 mg/L
MI	872 ± 29.13^a^	661.60 ± 36.60^b^	553.10 ± 34.67^c^	456.80 ± 22.74^d^	377.60 ± 28.79^e^
MN	0.38 ± 0.29^e^	17.90 ± 2.69^d^	26.30 ± 2.90^c^	35.90 ± 2.60^b^	45.50 ± 2.90^a^
SC	0.14 ± 0.09^e^	27.70 ± 0.79^d^	44.40 ± 2.55^c^	56.20 ± 3.85^b^	73.30 ± 5.14^a^
VC	0.40 ± 0.33^e^	20.70 ± 2.79^d^	28.40 ± 2.07^c^	37.10 ± 3.28^b^	46.70 ± 2.36^a^
FRG	0.00 ± 0.00^e^	15.50 ± 2.55^d^	23.50 ± 2.01^c^	30.50 ± 2.42^b^	39.50 ± 2.76^a^
UDC	0.00 ± 0.00^e^	7.90 ± 2.49^d^	14.50 ± 1.78^c^	20.20 ± 2.35^b^	29.20 ± 2.15^a^
RP	0.00 ± 0.00^e^	4.50 ± 2.72^d^	11.30 ± 2.83^c^	19.10 ± 2.02^b^	26.60 ± 2.07^a^
B	0.00 ± 0.00^e^	3.70 ± 2.11^d^	9.20 ± 1.48^c^	16.10 ± 2.47^b^	22.90 ± 2.13^a^
MA	0.00 ± 0.00^e^	2.10 ± 1.45^d^	7.20 ± 1.75^d^	13.90 ± 2.60^b^	18.10 ± 1.79^a^

The means shown with different superscript letters (a–e) in the same line are statistically significant (p < 0.05).

*MA* metaldehyde, *MI* mitotic index, *MN* micronucleus, *SC* sticky chromosome, *VC* vagrant chromosome, *FRG* fragments, *UDC* unequal distribution of chromatin, *RP* reverse polarization, *B* bridge, *MA* multipolar anaphase.

**Figure 2:**
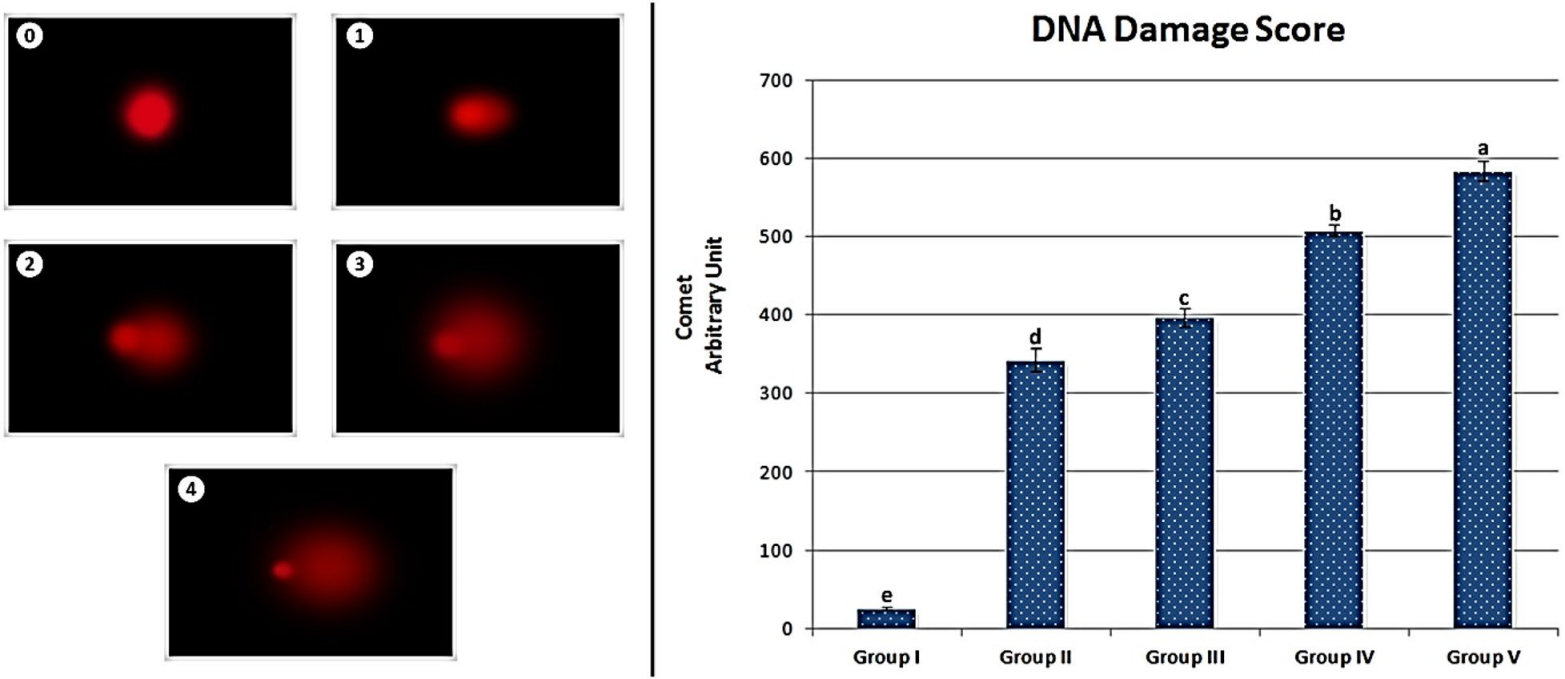
The effect of metaldehyde treatment on *A.* *cepa* L. root tip cell nuclei (0: no damage, 1: low damage, 2: moderate damage, 3: high damage, 4: extreme damage. Group I: control, Group II: 20 mg/L metaldehyde, Group III: 40 mg/L metaldehyde, Group IV: 100 mg/L metaldehyde, Group V: 200 mg/L metaldehyde).

The effects of metaldehyde treatment on DNA fragmentation in root tip cells of *A.* *cepa* L. are given in Fig. 2. As evident from the data, metaldehyde treatment caused DNA fragmentation in root tip cells of *A.* *cepa* L. While the average DNA damage score was 24.17 ± 4.07 in the control group, a sharp increase occurred in the MA-20 mg/L group and the average DNA fragmentation score was 341.67 ± 34.52. In the MA-40 mg/L group, the DNA fragmentation score increased to 395.83 ± 28.97. The DNA damage score was determined as 507.17 ± 18.64 in the MA-100 mg/L group and 583.50 ± 30.37 in the MA-200 mg/L group. As the metaldehyde doses increased, the DNA fragmentation score also increased. Differences in DNA damage scores between groups were statistically significant (p < 0.05).

**Figure 3:**
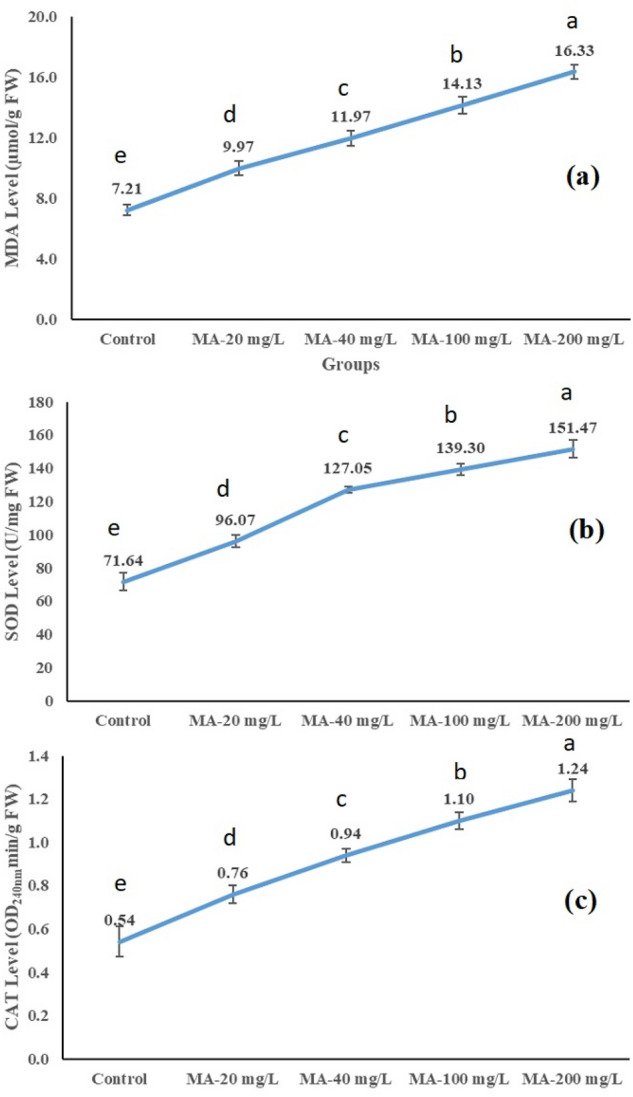
The effect of metaldehyde treatments on biochemical parameters.

Figure 3 depicts metaldehyde-induced changes in MDA level and SOD and CAT activities. Metaldehyde application gradually increased MDA level (Fig. 3a) as well as the activities of SOD (Fig. 3a) and CAT enzymes (Fig. 3c). The highest dose (200 mg/L) of metaldehyde caused the highest statistical increase in all biochemical parameters when compared to those in control (p < 0.05). In the MA-200 mg/L group, MDA levels and activities of SOD and CAT enzymes were increased by metaldehyde more than twice of their control group counterparts. Toxic compounds can affect the activities of antioxidant enzymes such as SOD and CAT, which indicate the toxicity level and tolerance capacity of plants^49^. CAT and SOD enzymes are important parts of the plant antioxidant system, and their increased levels were indicators of the elevated level of reactive oxygen species (ROS) in *A.* *cepa* due to pesticides^17,50^. Similarly, excessive ROS production increases membrane lipid peroxidation in plants, which can be measured by MDA content^51^. In the present study, the gradual increase in MDA level indicated increased membrane damage caused by metaldehyde-induced ROS accumulation. Similarly, increased CAT and SOD enzymes due to the plant's activated oxidative defense system indicated an increased ROS level caused by metaldehyde. Increased levels of ROS can induce serious harmful effects, including severe DNA damage^52^. In this context, our biochemical parameters showing ROS accumulation are compatible with genotoxic and growth parameters. The biochemical parameters of our study revealed that metaldehyde caused oxidative stress, which triggered serios cell membrane damage and genotoxic injuries. The antioxidant defense system containing SOD and CAT enzymes was activated against metaldehyde, but it could not eliminate all of the undesirable effects.

**Figure 4:**
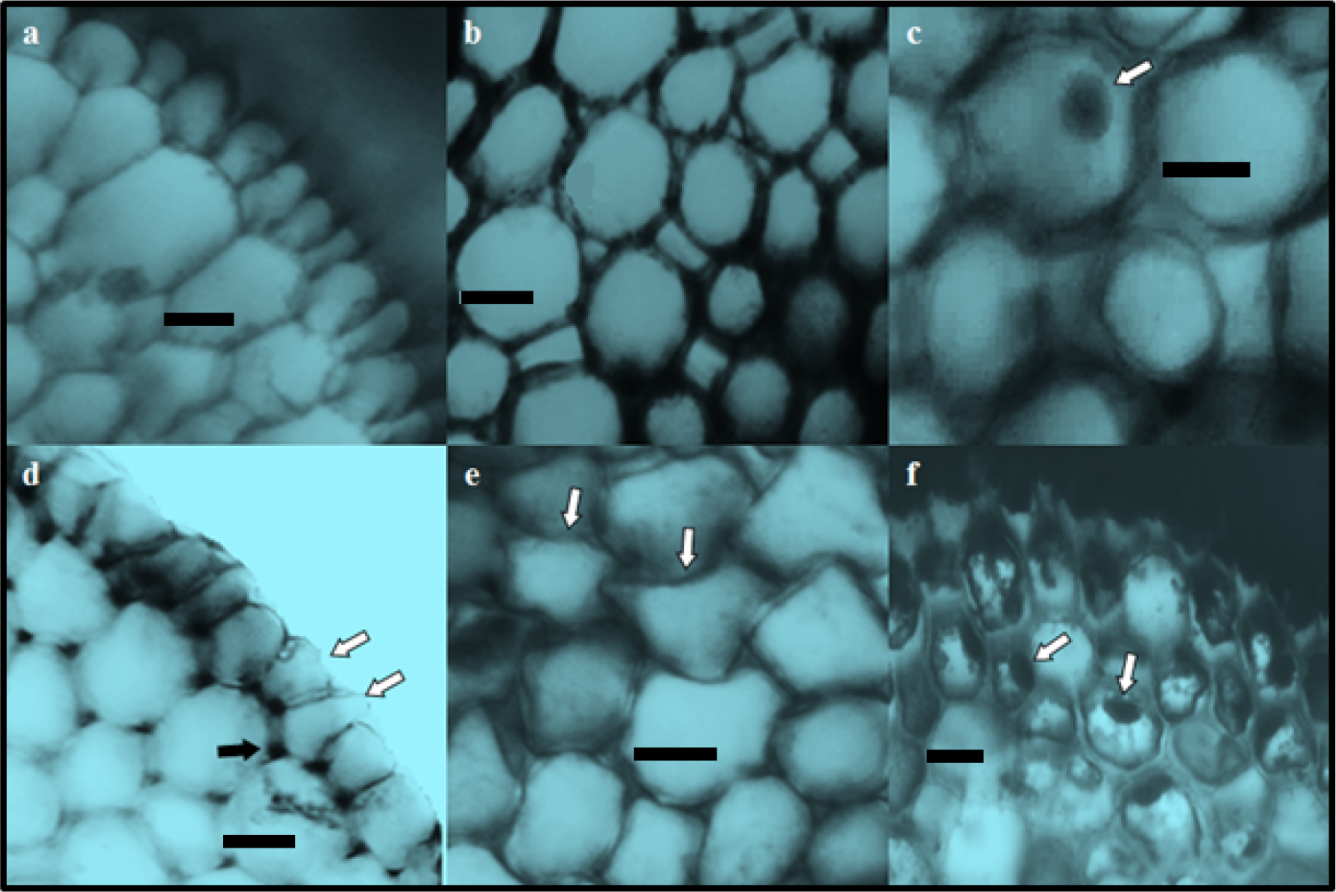
Anatomical damages induced by metaldehyde in root tip meristem cells. Normal appearance of epidermis cells (**a**), normal appearance of cortex cells (**b**), normal appearance of cell nucleus—oval, (**c**) epidermis cell damage—white arrows, thickening of the cortex cell wall—black arrow (**d**), cortex cell damage (**f**), flattened cell nucleus (**g**) (scale bar = 50 μm).

Anatomical damage induced by metaldehyde exposure is shown in Table 3 and Fig. 4. No damage was observed in the meristem cells of the roots of the control group. Metaldehyde application caused anatomical damage in root meristem cells in the form of epidermal cell damage, cortex cell damage, thickening of the cortex cell wall and flattened cell nucleus, the severity of which depends on the dose. Although there is no study in the literature investigating the anatomical changes caused by metaldehyde in plant root tip cells, there are some studies investigating the anatomical changes induced by other pesticides in *A.* *cepa* root tip meristem cells. For example, Tütüncü et al.^53^ determined that methiocarb administration at 2.5, 5.0 and 7.5 mg/L doses caused necrosis, epidermis cell deformation and thickening of the cortex cell wall in *A.* *cepa* root meristem cells. They also found that the severity of these damages was related to the methiocarb dose. Macar et al.^54^ reported that the application of 125 mg/L fenpyroximate caused deformation in the epidermis, flattening of the cell nucleus and damage in the conduction tissue in *A.* *cepa* root tip meristem cells. Kalefetoğlu Macar et al.^15^ observed that the administration of a 100 mg/L dose of diniconazole promoted epidermis cell deformation, thickening of the cortex cell wall, flattened cell nuclei and unclearly vascular tissue damage in *A.* *cepa* root tip cells. In this study, it is thought that the damage to root meristem cells as a result of exposure to metaldehyde is caused by the defense mechanisms developed by the cells to prevent metaldehyde from being absorbed. In microscopic examinations, increases were observed in the number and sequence of epidermis and cortex cells in metaldehyde-treated groups. These increases are a defense mechanism carried out by the plant in order to prevent metaldehyde uptake into the cell. However, since these increases increase the contact of the cells with each other and the mechanical pressure, deformities in the epidermis and cortex cells and the nucleus of these cells are inevitable. The information in the literature that plants have developed some chemical (synthesis of alkaloids, terpenoids, phenolic compounds, etc.) and morphological (increase in the number of trichomes, leaves, roots, cells and layers, etc.) defense mechanisms to restrict the entry of pesticides into the cell confirms this idea^29,55,56^.

**Table 3:** Anatomical damage induced by metaldehyde.

Groups	ECD	CCD	TCC	FCN
Control	−	−	−	−
MA-20 mg/L	+	+	+	+
MA-40 mg/L	++	+	+	++
MA-100 mg/L	+++	++	++	+++
MA-200 mg/L	+++	+++	+++	+++

*MA* metaldehyde, *ECD* epidermis cell deformation, *CCD* cortex cell deformation, *TCC* thickening of the cortex cell wall, *FCN* flattened cell nucleus. (−): no damage, (+): little damage, (++): moderate damage, (+++): severe damage.

## Conclusion

Metaldehyde has been widely used as a successful mollucide, but there is insufficient information about its effects on non-target organisms. *A.* *cepa* is a well-known and accomplished model plant for toxicity studies. A versatile research procedure including physiological, cytogenetic, biochemical and anatomical parameters was carried out to reveal the toxic effects of metaldehyde on *A.* *cepa*.

It was determined that metaldehyde application caused genotoxicity and oxidative stress in *A.* *cepa* depending on the dose. Reduced MI and increased DNA fragmentation, MN and CAs frequencies clearly indicated a metaldehyde-induced genotoxicity. Metaldehyde triggered oxidative damage and promoted ROS production, acting as a genotoxicity enhancer. In addition, metaldehyde exposure promoted a decrease in physiological parameters and anatomical damage to the roots. The EC_50_ value for metaldehyde on *A.* *cepa* was determined as 50 mg/L. In the literature, there is no comprehensive study investigating the toxicity of metaldehyde in plants. Therefore, this study is the most comprehensive study investigating all aspects of the physiological, cytogenetic, biochemical and anatomical effects of metaldehyde in *A.* *cepa.* The results of this study highlighted the need for new and detailed studies on the undesirable effects of metaldehyde on non-target organisms, including humans.

## Data Availability

All data are available in the main text or in the supplementary information.
